# Vitamin D Supplementation in Influenza and COVID-19 Infections Comment on: “Evidence that Vitamin D Supplementation Could Reduce Risk of Influenza and COVID-19 Infections and Deaths” *Nutrients* 2020, *12*(4), 988

**DOI:** 10.3390/nu12061626

**Published:** 2020-06-01

**Authors:** Chia Siang Kow, Muhammad Abdul Hadi, Syed Shahzad Hasan

**Affiliations:** 1 School of Postgraduate Studies, International Medical University, Kuala Lumpur 57000, Malaysia; Kowchiasiang@student.imu.edu.my; 2 School of Pharmacy, Institute of Clinical Sciences, University of Birmingham, Birmingham B15 2TT, UK; m.a.hadi@bham.ac.uk; 3 School of Applied Sciences, University of Huddersfield, Huddersfield HD1 3DH, UK

## Abstract

There is an ongoing debate on the use of vitamin D supplementation in reducing the risk of influenza and COVID-19 infections and deaths. A recently published article highlights a relationship between vitamin D supplementation and reduced risk of COVID-19 and influenza. This comment aims to discuss the evidence on the use of Vitamin D in people who are at risk of developing COVID-19, focusing on safety issues of the Vitamin D supplementation.

We read with interest the review article entitled “Evidence that Vitamin D supplementation could reduce risk of Influenza and COVID-19 infections and deaths” by Grant et al., recently published in *Nutrients* [1].

The authors’ work on reviewing possible mechanisms through which vitamin D supplementation could reduce risk of various respiratory infections, including the COVID-19, must be applauded as it has provided a rationale for conducting well-designed clinical studies to evaluate the effectiveness of vitamin D in reducing the risk of COVID-19 infection.

However, we are rather concerned with the authors’ recommendation that people at risk of COVID-19 should consider “taking 10,000 IU/d of vitamin D3 for a few weeks to rapidly raise 25(OH)D concentrations, followed by 5000 IU/d to reduce the risk of infection”. We believe that the authors’ recommendation of using a high dose of vitamin D supplementation is inappropriate as there is no robust clinical evidence to support such claims.

The authors have conveniently ignored the results of some key clinical studies evaluating the effectiveness of vitamin D supplementation in reducing the risk of developing respiratory tract infections (RTIs). A meta-analysis of 15 randomized controlled trials investigating the effectiveness of vitamin D supplementation in reducing the risk of developing RTIs among healthy individuals found no significant risk reduction [2]. However, some may argue that heterogeneity among included studies was high and, therefore, should not be used to deny the potential benefits of vitamin D supplementation. Using individual patient data (IPD), when available for meta-analysis, may be a better approach to overcome inconsistencies at the trial level. 

However, the authors did include results of an IPD-based meta-analysis (including of 25 randomized, double-blind, placebo-controlled trials) evaluating the effectiveness of supplementation with vitamin D3 or vitamin D2 with prespecified acute RTIs as an outcome. The IPD-based meta-analysis did find 12% reduction in the odds of acquiring acute respiratory tract infection, however, further analysis reported no significant benefit of vitamin D supplementation in population with levels of vitamin D ≥10 ng/mL [3]. A significant benefit was also absent in population receiving a daily dose equivalent of ≥800 IU (20 μg) vitamin D supplementation.

Although high-dose vitamin D3 was not found to increase the risk of kidney stone or hypercalcemia [4], it is not devoid of side effects, as a randomized clinical trial observed significant lower radial bone and tibial bone mineral density with 3 year treatment of vitamin D at a dose of 10,000 IU/d [5].

In conclusion, the efficacy of high-dose supplementation of vitamin D_3_ in reducing risk of COVID-19 infection is mere extrapolation of currently available evidence, which is often conflicting, on the effectiveness of vitamin D3 in reducing risk of other respiratory tract infections. Given the possible negative impact on bone mineral density with high-dose vitamin D3, it is probably wise to wait for the results of ongoing clinical trials that are registered to explore the relationship between vitamin D and COVID-19 [6,7]. 

## References

[1] Grant W.B., Lahore H., McDonnell S.L., Baggerly C.A., French C.B., Aliano J.L., Bhattoa H.P. (2020). Evidence that vitamin D supplementation could reduce risk of influenza and covid-19 infections and deaths. Nutrients.

[2] Vuichard Gysin D., Dao D., Gysin C.M., Lytvyn L., Loeb M. (2016). Effect of vitamin D3 supplementation on respiratory tract infections in healthy individuals: A systematic review and meta-analysis of randomized controlled trials. PLoS ONE.

[3] Martineau A.R., Jolliffe D.A., Hooper R.L., Greenberg L., Aloia J.F., Bergman P., Dubnov-Raz G., Esposito S., Ganmaa D., Ginde A.A. (2017). Vitamin D supplementation to prevent acute respiratory tract infections: Systematic review and meta-analysis of individual participant data. BMJ (Clinical research ed.).

[4] Malihi Z., Lawes C.M.M., Wu Z., Huang Y., Waayer D., Toop L., Khaw K.T., Camargo C.A., Scragg R. (2019). Monthly high-dose vitamin D supplementation does not increase kidney stone risk or serum calcium: Results from a randomized controlled trial. Am. J. Clin. Nutr..

[5] Burt L.A., Billington E.O., Rose M.S., Raymond D.A., Hanley D.A., Boyd S.K. (2019). Effect of high-dose vitamin D supplementation on volumetric bone density and bone strength: a randomized clinical trial. JAMA.

[6] Clinical Trial [Trial No: IRCT20200324046850N1]. https://irct.ir/trial/46732.

[7] Clnical Trial [Trial No: ChiCTR2000031163]. http://www.chictr.org.cn/showprojen.aspx?proj=51390.

